# Apple F-Box Protein MdMAX2 Regulates Plant Photomorphogenesis and Stress Response

**DOI:** 10.3389/fpls.2016.01685

**Published:** 2016-11-17

**Authors:** Jian-Ping An, Rui Li, Feng-Jia Qu, Chun-Xiang You, Xiao-Fei Wang, Yu-Jin Hao

**Affiliations:** National Key Laboratory of Crop Biology, National Research Center for Apple Engineering and Technology, College of Horticulture Science and Engineering, Shandong Agricultural UniversityTai’an, China

**Keywords:** MAX2, strigolactones, photomorphogenesis, stress responses, apple

## Abstract

MAX2 (MORE AXILLARY GROWTH2) is involved in diverse physiological processes, including photomorphogenesis, the abiotic stress response, as well as karrikin and strigolactone signaling-mediated shoot branching. In this study, MdMAX2, an F-box protein that is a homolog of *Arabidopsis* MAX2, was identified and characterized. Overexpression of *MdMAX2* in apple calli enhanced the accumulation of anthocyanin. Ectopic expression of *MdMAX2* in *Arabidopsis* exhibited photomorphogenesis phenotypes, including increased anthocyanin content and decreased hypocotyl length. Further study indicated that MdMAX2 might promote plant photomorphogenesis by affecting the auxin signaling as well as other plant hormones. Transcripts of *MdMAX2* were noticeably up-regulated in response to NaCl and Mannitol treatments. Moreover, compared with the wild-type, the *MdMAX2*-overexpressing apple calli and *Arabidopsis* exhibited increased tolerance to salt and drought stresses. Taken together, these results suggest that MdMAX2 plays a positive regulatory role in plant photomorphogenesis and stress response.

## Introduction

Apple tree (*Malus* × *domestica*) is one of the most important economic crops in the world and is used to provide both fruit and woods (Wang et al., 2015). The cultivation and yield of apple is frequently limited by various external environmental factors, such as light, temperature, and nutrients. Numerous genes are induced by external environmental factors to regulate plant growth and development (Xiong et al., 2002; Todaka et al., 2015). Therefore, it is necessary to study the functions of genes that are regulated by external environmental factors to improve crops’ adaptation to the variable environment.

Among the external environmental factors, light is one of the most important factors that acts as the primary energy source for photosynthesis as well as environmental signaling to affect plant physiology and morphology throughout the lifecycle (Shen et al., 2007; Xu et al., 2014). Depending on the different light conditions, plants exhibit two contrasting growth statuses: skotomorphogenesis in the dark and photomorphogenesis in the light. Skotomorphogenesis is characterized by elongated hypocotyls and closed cotyledons. In contrast, photomorphogenesis is characterized by shortened hypocotyls and open cotyledons (Von Arnim and Deng, 1996; Li K. et al., 2015). A suite of sensory photoreceptors, including cryptochromes (CRY), phytochrome (PHY), phototropins, and UV-B receptor UVR8, allow plants to monitor and respond to light signals and then to regulate plant growth and development (Chen et al., 2004; Rizzini et al., 2011).

In addition to the light, external stresses, such as soil salinity and drought, have become serious cues that limit the productivity and quality of crops (Zhu, 2007). High salinity in soil causes both ionic and osmotic stresses (Tian et al., 2015), and drought stress results in a decrease of water availability (Sequera-Mutiozabal et al., 2016). Currently, a cascade of signaling events is known to be involved in the response to stresses, such as second messengers and the MAPKs cascade. Among these response strategies, abscisic acid (ABA) plays an essential role in reporting environmental stimuli. Stomatal aperture, in particular, is regulated by ABA during the adaptive response to salt and drought stresses. When plants encounter salt or drought stress, ABA induces stomatal closure to maintain water (Yamaguchi-Shinozaki and Shinozaki, 2006; Savchenko et al., 2014).

Various phytohormones, such as cytokinin (CK), auxin, gibberellins (GAs), ethylene, and ABA, are known to cooperatively regulate plant growth and development (Kazan, 2015; Li K. et al., 2015; Schaller et al., 2015). Besides, strigolactones (SLs) have been identified as a new group of plant hormones that were originally known for their function of stimulating seed germination of root parasitic plants, such as *Orobanche* species and *Striga* (Wall et al., 1972; Waldie et al., 2014). Recent studies have demonstrated that SLs regulate various aspects of plant growth and development, such as inhibition of axillary bud outgrowth (Lazar and Goodman, 2006; Brewer et al., 2015), rhizosphere parasitic and symbiotic interactions (Cook et al., 1966; Akiyama et al., 2005), response to salt and drought stress (Van Ha et al., 2014), and modulation of root development (Koltai et al., 2010; Kapulnik et al., 2011; Sun et al., 2016). In SL signaling, four major genes have synergistic effects to regulate plant development, *MAX1. MAX3*/*CCD7. MAX4*/*CCD8*, and MORE AXILLARY GROWTH2 (MAX2). Among these, *MAX1. MAX3*, and *MAX4* are required for the biosynthesis of SLs (Turnbull et al., 2002; Booker et al., 2004); *MAX2* encodes an F-box leucine-rich repeat (LRR) protein, which is a component of the SCF (for SKP, Cullin, and F-box protein) complex of ubiquitin ligases (Stirnberg et al., 2002). MAX2 is suggested to be involved in the perception of SL signaling, and *Arabidopsis max2* mutants exhibit a phenotype of insensitivity to SLs (Ruyter-Spira et al., 2013).

MORE AXILLARY GROWTH2 is centrally involved in many important biological processes in plants. For example, previous studies have shown that MAX2 promotes photomorphogenic development in response to light (Shen et al., 2007, 2012) and enhances the tolerance to drought and salt stress; the *Arabidopsis max2* mutant shows hypersensitivity to drought and salt stress (Bu et al., 2014; Van Ha et al., 2014). Moreover, MAX2 plays a key role in SL-mediated shoot branching and root development (Bennett et al., 2006; Nelson et al., 2011; Mayzlish-Gati et al., 2012). So far, MAX2 has been cloned and functionally identified in *Arabidopsis* and rice as well as in pea and orange. However, little is known about the functions of MAX2 in apple.

In the present study, F-box protein MdMAX2 was cloned and functionally characterized in apple. Overexpression of *MdMAX2* increased anthocyanin accumulation in apple calli, while ectopic expression of *MdMAX2* in *Arabidopsis* increased anthocyanin accumulation and decreased hypocotyl length. Further study indicated that MdMAX2 promoted plant photomorphogenesis by regulating auxin signaling. Additionally, *MdMAX2* was induced by multiple hormones and abiotic stresses; overexpression of *MdMAX2* enhanced tolerance to salt and drought stress in transgenic apple calli and transgenic *Arabidopsis*.

## Materials and Methods

### Plant Materials and Growth Conditions

Apple (*Malus* × *domestica*) calli of the ‘Orin’ cultivar (WL) were used as wild-type (WL) and for genetic transformation and other analyses. The apple calli were grown on Murashige and Skoog (MS) medium supplemented with 0.5 mg⋅L^-1^ indole-3-acetic acid (IAA) and 1.5 mg⋅L^-1^ 6-benzylaminopurine (6-BA) at 24°C in the dark and were subcultured at 15 days intervals.

*Arabidopsis* ecotype Columbia (Col-0) plants were used in the study. The seeds were sown on MS medium after being treated for 4 days at 4°C. Seedlings were grown in an incubator at 22°C in long-day conditions (16-h-light/8-h-dark) under fluorescent lights (photon flux density about 46 umol s^-1^m^-2^).

### Sequence Alignment and Phylogenetic Analysis

To obtain the homologs of MdMAX2, BLASTP program^1^ was performed. Phylogenetic analysis was conducted in MEGA software version 5.0. For the phylogenetic tree construction, 1000 bootstrap replicates had been performed. The protein secondary structure of MdMAX2 was predicted using Simple Modular Architecture Research Tool (SMART) software^2^.

### Plasmid Construction and Genetic Transformation

The overexpression vectors *MdMAX2-GFP* and *MdMAX2-GUS* were constructed by inserting the DNA fragment of the *MdMAX2* open reading frame (ORF) into the transformed vectors pCAMBIA1300-GFP and pCAMBIA1300-GUS, respectively.

To generate *MdMAX2-GFP* and *MdMAX2-GUS* transgenic apple calli, the recombinant plasmids were transferred to *Agrobacterium* tumefaciens LBA4404. The transgenic calli were obtained according to the method described by An et al. (2015). Transgenic *Arabidopsis* plants were generated through the floral dip transformation method (Clough and Bent, 1998). Single-locus T-DNA insertional transgenic lines were selected for further characterization.

### Measurements of Anthocyanin

Total anthocyanin was extracted with the methanol-HCl method (Lee and Wicker, 1991). Apple calli or seedlings were placed in an anthocyanin extraction solution for 24 h at 4°C in the dark. The absorbance at 530, 620, and 650 nm was measured using a Uv-vis spectrophotometer (SHI-MADZU UV-2450, Kyoto, Japan). The anthocyanin content was normalized using the following formula: OD = (A530 – A620) - 0.1(A650 – A620). One unit of anthocyanin content was expressed as a change of 0.1 OD (unit × 10^3^ g^-1^ FW).

### Real-Time Quantitative PCR (qRT-PCR)

The transcription levels of *MdMAX2* were examined using specific primers MdMAX2(qRT)-F and MdMAX2(qRT)-R. ACTIN was used as the control. All of the primers used are shown in Supplementary Table S1. Each experiment was repeated at least three times. The experiments were based on the average of three parallel experiments.

### Hypocotyl Length Measurements

The hypocotyl lengths of at least 30 *Arabidopsis* seedlings that were grown vertically on MS medium in the light or dark were measured. The lengths of hypocotyls were measured with free software ImageJ^3^ (Bai et al., 2014). The experiments were based on the average of three parallel experiments.

### Gravitropism Assays

Seven-day-old seedlings were gravistimulated by a 135° rotation and monitored in a lightproof box equipped with a spectrum-enhanced camera (EOS035 Canon Rebel T3i); this was modified by Hutech technologies using a built-in, clear, wide-band, multi-coated filter, and operated using the EOS utility software. Embedded infrared light-emitting diodes (LEDs, 880 nm) dispensed light to illuminate the samples. The angles of the root tips were recorded after 5, 10, and 15 h and were analyzed with the ImageJ software. Eight to twelve roots per line were measured in three individual experiments (Löfke et al., 2015).

### Morphological Characterization of Roots

Root morphology was examined on MS medium solidified with 0.6% agar. Briefly, seeds were germinated on MS medium. Five-day-old seedlings were transferred to MS medium, and the plates were grown vertically for several days. The visible LR numbers were counted daily beginning on the transfer day, and pictures of the plates were taken. Digital images of the plants were used for image software (NIH)-based manual root length measurements. For LRPs and LRs of DR5, GUS plants from different genetic backgrounds were photographed or counted using HIROX’s KH-7700 digital microscope, and a classification of the LRP developmental stages was performed as previously described by Malamy and Benfey (1997).

### Root Hair Measurements

For root hair analyses, 4-day-old seedlings grown vertically on MS medium were used for the root hair measurements. Roots were examined with an Olympus BZX16 microscope. Quantification of the root hair length and number was performed using ImageJ^4^. In the experiments, three replicate experiments involving 30–40 root hairs were performed for each genetic background (Chai et al., 2016).

### Statistical Analysis

The statistical analysis was performed with appropriate methods using R (3.0.2) with the R Commander package. Differences were considered statistically significant when ^∗^*P* < 0.05 and ^∗∗^*P* < 0.01. All the results were based on the average of three parallel experiments.

## Results

### Molecular Cloning and Sequence Analysis of *MdMAX2*

**FIGURE 1:**
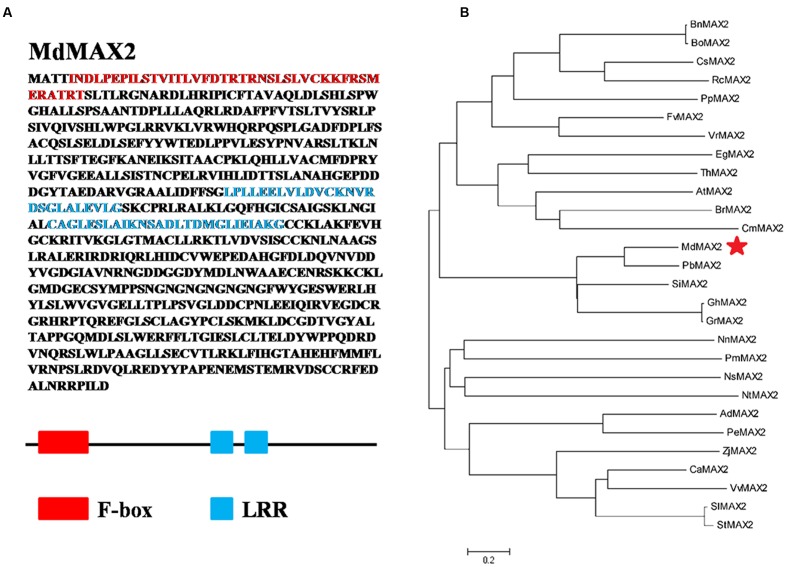
**Structure analysis of MdMAX2 and phylogenetic analysis of MAX2 proteins from 28 different plant species.** **(A)** Functional domains analysis of MdMAX2. The analysis was performed using SMART (http://smart.embl-heidelberg.de/smart/list_genomes.pl). Red represents the F-box domain (5–45 aa). Blue represents two LRR repeat domains (213–244 aa and 372–397 aa). **(B)** Phylogenetic analysis of MdMAX2 and the other 27 plants’ MAX2 protein sequences obtained from the NCBI database. The phylogenetic tree was constructed with MEGA 4.0. MdMAX2 is denoted by a red asterisk, and the scale bar indicates the branch length. CsMAX2: *Camelina sativa*, XP_010517801.1; BrMAX2: *Brassica rapa*, XP_009142077.1; BnMAX2: *Brassica napus*, XP_013687871.1; BoMAX2: *Brassica oleracea*, XP_013637137.1; ThMAX2: *Tarenaya hassleriana*, XP_010524067.1; RcMAX2: *Ricinus communis*, XP_002528551.1; PbMAX2: *Pyrus bretschneideri*, XP_009370527.1; PmMAX2: *Prunus mume* XP_016649047.1; PpMAX2: *Prunus persica*, XP_007214985.1; FvMAX2: *Fragaria vesca*, XP_011466958.1; ZjMAX2: *Ziziphus jujuba*, XP_015881238.1; VvMAX2: *Vitis vinifera*, XP_010657042.1; GrMAX2: *Gossypium raimondii*, XP_012437380.1; CmMAX2: *Cucumis melo*, XP_008455299.1; GhMAX2: *Gossypium hirsutum*, AIM41254.1; VrMAX2: *Vigna radiata*, XP_014493834.1; EgMAX2: *Eucalyptus grandis*, XP_010035860.1; NtMAX2: *Nicotiana tabacum*, XP_016491087.1; AdMAX2: *Arachis duranensis*, XP_015936416.1; PeMAX2: *Populus euphratica*, XP_011033739.1; SiMAX2: *Sesamum indicum*, XP_011083007.1; StMAX2: *Solanum tuberosum*, XP_006348874.1; SlMAX2: *Solanum lycopersicum*, XP_004243284.1; NsMAX2: *Nicotiana sylvestris*, XP_009766305.1; CaMAX2: *Capsicum annuum*, XP_016541573.1; NnMAX2: *Nelumbo nucifera*, XP_010279132.1.

To isolate the full-length cDNA sequence of *MdMAX2* corresponding to the NCBI EST database, RT-PCR was conducted using cDNA templates from *in vitro* ‘Gala’ apple tissue cultures. *MdMAX2* (MDP0000466825), a gene containing a 2163-bp ORF, was obtained. The *MdMAX2* gene encoded a protein of 720 amino acid residues. Amino acid sequence analysis showed that MdMAX2 contained an F-box motif in its N-terminus and two LRR repeat motifs in its C-terminus that were highly conserved in a variety of plant species (**Figure 1A**; **Supplementary Figure S1**). To analyze the phylogenetic relationship between MdMAX2 and MAX2 proteins from other plant species, a phylogenetic tree analysis of 28 plants’ MAX2 proteins was constructed using the MEGA 5.0 software. As shown in **Figure 1B**, the phylogenetic tree revealed that the MAX2 proteins were divided into five main clusters, and MdMAX2 formed a close cluster with PbMAX2, SiMAX2, GhMAX2, and GrMAX2. The results suggested that MdMAX2 was most closely related to the *Pyrus bretschneideri* PbMAX2 protein. Additionally, qRT-PCR showed that *MdMAX2* was expressed in all organs, with its highest expression levels evident in the roots and leaves (**Supplementary Figure S3A**).

### MdMAX2 Is a Positive Regulator of Anthocyanin Accumulation in Apple

**FIGURE 2:**
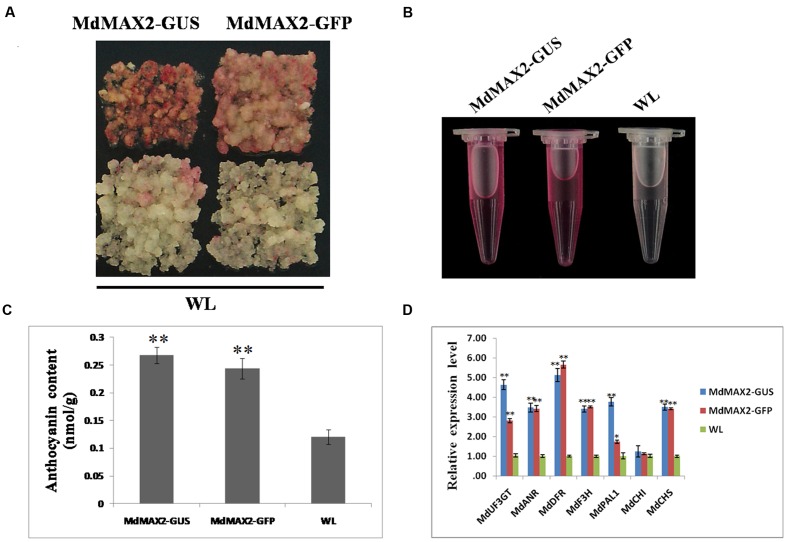
**Overexpression of *MdMAX2* increases the anthocyanin content in apple calli.** **(A–C)** Colors and anthocyanin contents in the wild-type control (WT) and the transgenic calli (MdMAX2-GFP and MdMAX2-GUS) grown on a medium with high light treatment. **(D)** qRT-PCR showed that MdMAX2 affected the expression of genes (*MdDFR. MdUF3GT. MdF3H. MdCHS. MdCHI. MdANR. MdPAL1*) involved in anthocyanin biosynthesis in apples. Values are the mean ± SD of three replica experiments, and asterisks denote Student’s test significance compared with the wild-type plants: ^∗^*P* < 0.05; ^∗∗^*P* < 0.01.

To study the functions of MdMAX2, transgenic calli of overexpressing *MdMAX2* that introduced *35S::MdMAX2-GUS* and *35S::MdMAX2-GFP* into the ‘Orin’ cultivar (WL) were constructed (**Supplementary Figure S2A**). Additionally, two independent lines (MdMAX2-GUS and MdMAX2-GFP) were selected for further investigation. Previous studies have shown that *Arabidopsis* MAX2 functions as a positive regulator of photomorphogenesis (Shen et al., 2007, 2012). Furthermore, MAX1, a structural gene that regulates SL biosynthesis, is proved to be a positive regulator in the flavonoid pathway (Lazar and Goodman, 2006). Anthocyanin biosynthesis is considered to be an important indicator of light photomorphogenesis. To examine whether *MdMAX2* also regulates anthocyanin accumulation in apple, transgenic apple calli were used for coloration assays. As shown in **Figure 2A**, both *MdMAX2* overexpression calli (MdMAX2-GUS and MdMAX2-GFP) looked significantly redder than wild-type (WL) under high light conditions. The anthocyanin extracting solution (**Figure 2B**) and spectrophotometric analysis (**Figure 2C**) demonstrated that overexpression of *MdMAX2* accumulated more anthocyanin. Meanwhile, the expression levels of flavonoid structural genes in the MdMAX2-GUS, MdMAX2-GFP, and WL were analyzed by qRT-PCR. The results showed that the expression levels of anthocyanin biosynthesis-related genes, including *MdUF3GT. MdANR. MdDFR. MdF3H. MdPAL. MdCHI*, and *MdCHS*, were significantly up-regulated (**Figure 2D**), which demonstrated that MdMAX2 functioned as a positive regulator in anthocyanin accumulation in apple.

### MdMAX2 Functions as a Positive Regulator of Photomorphogenesis in *Arabidopsis*

**FIGURE 3:**
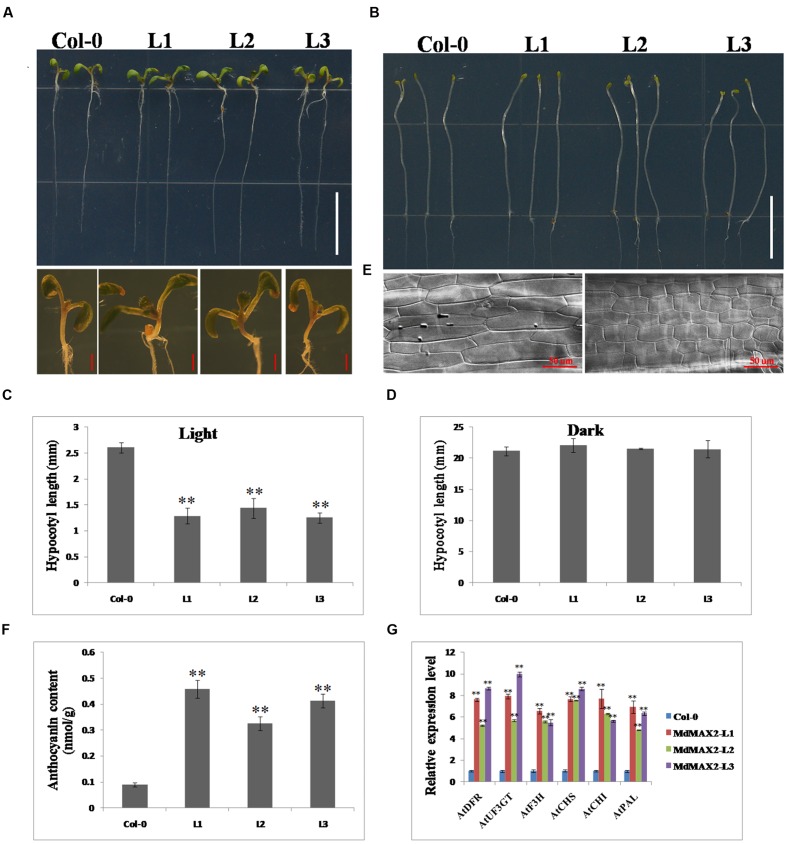
**Overexpression of *MdMAX2* positively regulates photomorphogenesis in *Arabidopsis*.** Seven-day-old wild-type seedlings (Col-0) and three MdMAX2 overexpression lines (L1, L2, and L3) grew under fluorescent light conditions **(A)** or dark conditions. Bars = 1 cm. **(B)**. In **(A)**, the panel below shows the hypocotyls that were grown in the light. Bars = 1 mm. **(C,D)** Hypocotyl length quantifications of Col-0 and MdMAX2 transgenic lines (L1, L2, and L3). **(E)** Hypocotyl cells in wild-type (Col-0) and *MdMAX2* transgenic lines (L1). The samples were visualized using a microscope (LSM 5 Exciter, Carl-Zeiss). **(F)** Comparison of anthocyanin content of overexpression lines with Col-0. **(G)** qRT-PCR showed that MdMAX2 affected the expression of genes (*AtDFR. AtUF3GT. AtF3H. AtCHS. AtCHI. AtPAL1*) involved in anthocyanin biosynthesis in *Arabidopsis*. Values are the mean ± SD of three replica experiments, and asterisks denote Student’s test significance compared with the wild-type plants: ^∗^*P* < 0.05; ^∗∗^*P* < 0.01.

To further investigate the functions of MdMAX2, the plasmid carrying the *35S::MdMAX2-GFP* was transformed into *A. thaliana* ecotype Columbia (Col-0) plants. After repeated selection on selection medium as well as PCR detection for the presence of the *MdMAX2* transgenic *Arabidopsis*, three independent lines (MdMAX2-L1, MdMAX2-L2, and MdMAX2-L3) were selected for the subsequent analysis (**Supplementary Figure S2B**). Interestingly, under normal light growth conditions, all three overexpression lines (L1, L2, and L3) exhibited a shorter hypocotyl than the wild-type (Col-0; **Figures 3A,C**). However, no significant differences in hypocotyl elongation were detected under continuous dark conditions (**Figures 3B,D**). Additionally, the overexpressing *MdMAX2* seedlings (MdMAX2-L1) exhibited shorter hypocotyl cells than those in Col-0 seedlings (**Figure 3E**).

The anthocyanin contents in Col-0 and overexpression lines were also compared. As shown in **Figures 3F–G**, all three overexpression lines accumulated significantly more anthocyanin than Col-0 under high light growth conditions, indicating that *MdMAX2* enhanced anthocyanin accumulation in *Arabidopsis*, which is similar to the function of *MdMAX2* in apple. The expression levels of anthocyanin biosynthesis-related genes, including *AtDFR. AtUF3GT. AtF3H. AtCHS. AtCHI*, and *AtPAL*, were also detected. As expected, the results showed that these anthocyanin biosynthesis-related genes had similar expression patterns in transgenic *Arabidopsis* within transgenic apple calli. These results showed that ectopic expression of *MdMAX2* in *Arabidopsis* shortened the hypocotyl length and increased the anthocyanin content, suggesting the possibility that MdMAX2 functioned as a positive regulator of photomorphogenesis in *Arabidopsis*.

### *MdMAX2* Regulates Photomorphogenesis Particularly through Influencing Auxin Transport and Distribution

**FIGURE 4:**
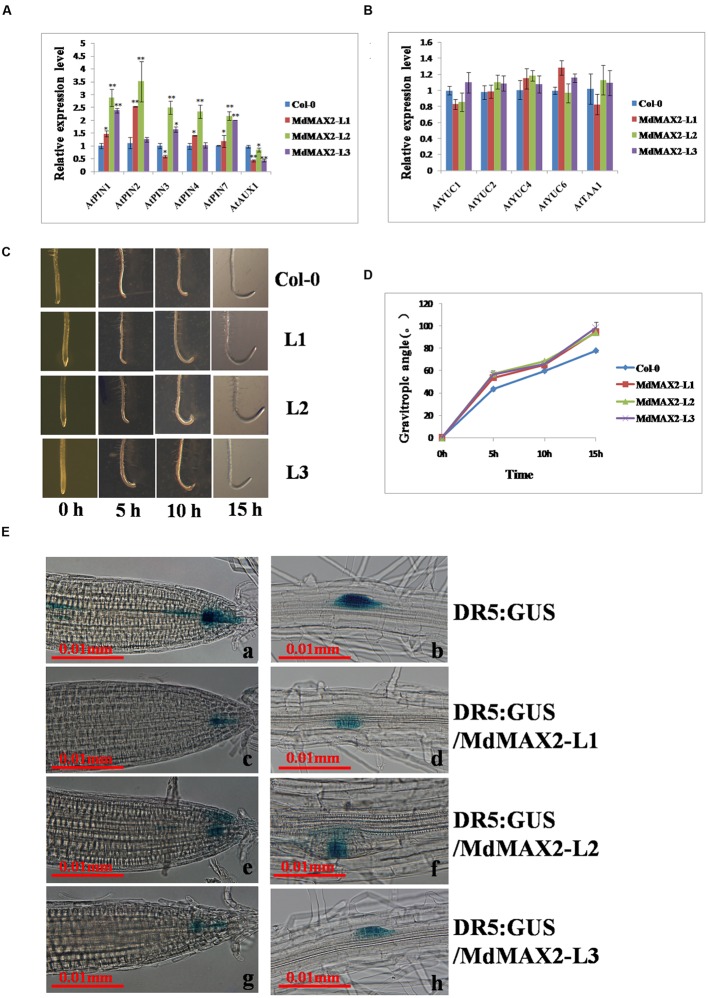
**MdMAX2 influences auxin signaling.** **(A–B)** Relative expression levels of auxin transport genes (*AtAUX1. AtPIN1. AtPIN2. AtPIN3. AtPIN4. AtPIN7*) and auxin biosynthesis genes (*AtYUC1. AtYUC2. AtYUC4. AtYUC6. AtTAA1*) in the roots of 9-day-old plants. The expression levels of each gene in the wild-type (Col-0) were set at 1. **(C–D)** Kinetics assays of the gravitropic response after 135° gravistimulation. The angle of every seedling was measured with respect to the initial (old) gravity vector after 5, 10, and 15 h. **(E)** Expression of *DR5: GUS* (*N* = 20 plants) in two different stages of the primary root tip (a, c, e, g), lateral root primordium (b, d, f, h) of wild-type plants, and *MdMAX2*-overexpression plants. Six-day-old plants that grew on Murashige and Skoog (MS) medium were used for GUS staining for 6 h. Values are the mean ± SD of three replica experiments, and asterisks denote Student’s test significance compared with the wild-type plants: ^∗^*P* < 0.05; ^∗∗^*P* < 0.01.

Numerous investigations have determined that certain level of auxin inhibited anthocyanin biosynthesis. Additionally, previous work has shown that auxin affected hypocotyl elongation (Gray et al., 1998). The above-mentioned evidences resulted in a hypothesis that the anthocyanin accumulation and hypocotyl elongation might be relative to auxin. To verify whether MdMAX2-mediated photomorphogenesis throught auxin, the expression levels of the auxin transport genes and auxin biosynthesis genes were analyzed. As expected, the results showed that the expression levels of auxin efflux carrier genes, including *AtPIN1. AtPIN2. AtPIN3. AtPIN4. AtPIN7*, were clearly increased in transgenic plants relative to wild-type seedlings. In contrast, the expression levels of auxin influx carrier genes, *AtAUX1*, were clearly decreased (**Figure 4A**). Subsequently, gravitropic root growth was investigated; and the results suggested that overexpression of *MdMAX2* had an impact on gravitropic root growth (Figures. 4C,D). Besides, the ARF-related genes (ARF1, ARF2, and ARF5) were significantly increased in the transgenic *Arabidopsis* (**Supplementary Figure S3D**).

Meanwhile, the expression levels of the auxin biosynthesis genes, including *AtYUC1. AtYUC2. AtYUC4. AtYUC6*,*AtTAA1. AtCYP798B2*, and *AtCYP798B3* showed no significant difference between wild-type and transgenic lines (**Figure 4B**; **Supplementary Figure S3D**). In addition, the auxin-responsive *DR5::GUS* marker line was crossed with the *MdMAX2*-overexpressing background lines to examine the endogenous auxin distribution in the roots. The histochemical staining results showed that expression levels of the *DR5::GUS* auxin response reporter protein in the transgenic lines (L1, L2, and L3) were noticeably reduced (**Figure 4E**), despite the unaltered expression levels of auxin biosynthesis genes (**Figure 4B**). This was possibly due to the feedback regulation of auxin. Shen et al. (2012) suggested that multiple hormone pathways contributed to *MAX2*-mediated photomorphogenesis, and our study also found that MdMAX2 responded to various hormones and stress conditions (**Supplementary Figure S3B**). Based on these facts, it is suggested that MdMAX2 might promote the photomorphogenesis particularly through mediating auxin as well as multiple hormones in apple.

### Overexpression of *MdMAX2* in *Arabidopsis* Enhanced the Tolerance to Salt Stress

**FIGURE 5:**
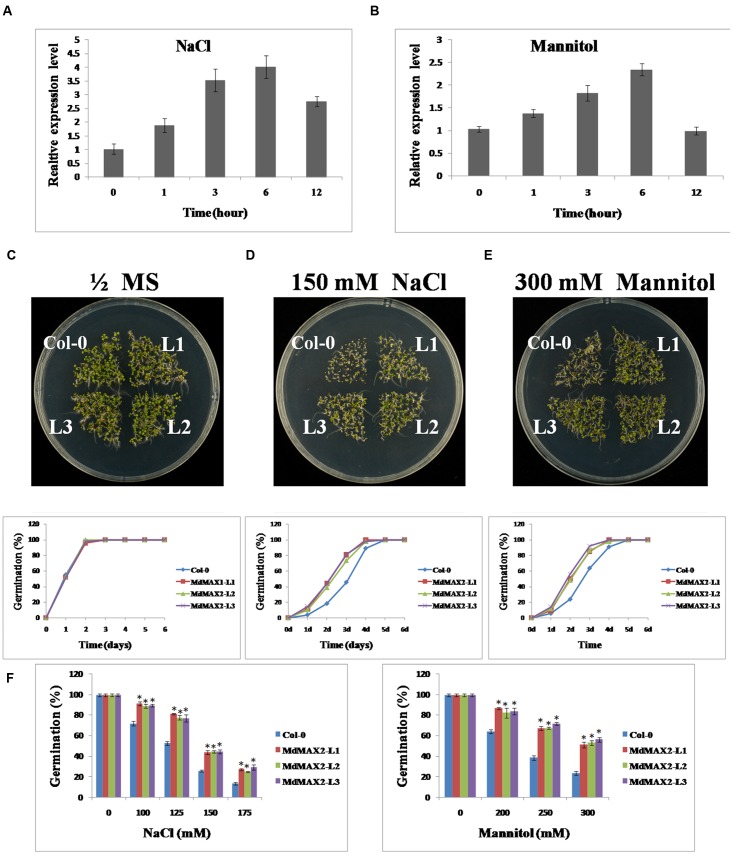
**MdMAX2 enhances *Arabidopsis* resistance to salt and osmotic stresses during seed germination.** **(A–B)** qRT-PCR analyses of *MdMAX2* in ‘Gala’ tissue cultures under salt and mannitol treatments. Twenty-day-old ‘Gala’ tissue cultures were treated with 150 mM NaCl and 300 mM Mannitol. Tissues were collected at different time intervals, and RNA was isolated to make the first strand of cDNA. Expression levels of *MdMAX2* were monitored by real-time PCR. **(C–E)** Salt and osmotic stress sensitivity of the wild-type and 35S::*MdMAX2* plants during the seed germination stage. Seeds of the indicated genotypes were sown on half-strength MS medium with 150 mM NaCl or 300 mM mannitol. **(F)** Germinating percentages of different-genotype seeds grown for 2 days after stratification on half-strength MS medium supplemented with different concentrations of NaCl (left panel) and mannitol (right panel). Values are the mean ± SD of three replica experiments, and asterisks denote Student’s test significance compared with the wild-type plants: ^∗^*P* < 0.05; ^∗∗^*P* < 0.01.

Many evidences have demonstrated that the F-box protein MAX2 plays an important role in stress response. The *max2* mutant seedlings exhibited a phenotype of hypersensitivity to salt and drought stresses (Bu et al., 2014; Van Ha et al., 2014). To evaluate the possibility of stress tolerance of the MdMAX2 protein in apple, qRT-PCR analyzed the expression levels of *MdMAX2* after NaCl and Mannitol treatments. As shown in **Figures 5A,B**, *MdMAX2* expression was induced by the NaCl and Mannitol treatments, indicating that MdMAX2 might play a vital role in the response to salinity and osmotic stresses.

To evaluate the stress tolerance of the MdMAX2 protein in plants, transgenic *Arabidopsis* were used for a series of tolerance assays. First, seed germination assays were performed. The seeds of Col-0 and transgenic *Arabidopsis* were germinated on 1/2 MS medium containing NaCl (0, 100, 125, 150, and 175 mM) and different concentrations of Mannitol (0, 200, 250, and 300 mM). The analysis showed that there were no obvious differences in the rates of the germination when seeds were grown on normal 1/2 MS medium (**Figure 5C**). However, in the presence of 150 mM NaCl and 300 mM Mannitol, the *35S::MdMAX2* transgenic plants showed much higher germination rates than Col-0 (**Figures 5B,C**). After 2 days on 100 mM NaCl, there was a germination ratio of approximately 90% for the transgenic seeds compared with approximately 70% for the wild-type seeds. When grown on the 1/2 MS medium containing 175 mM NaCl for 2 days, only 10% of the wild-type seeds germinated, compared to 20% of the *35S::MdMAX2* transgenic seeds (**Figure 5F**, left panel). Similar to the findings with mannitol, our studies demonstrated that osmotic stress inhibited seed germination of the wild-type plants much more than the *35S::MdMAX2* transgenic *Arabidopsis* (**Figure 5F**, right panel). These results suggested that the overexpression of *MdMAX2* enhanced the tolerance to salt and osmotic stresses at seed germination.

**FIGURE 6:**
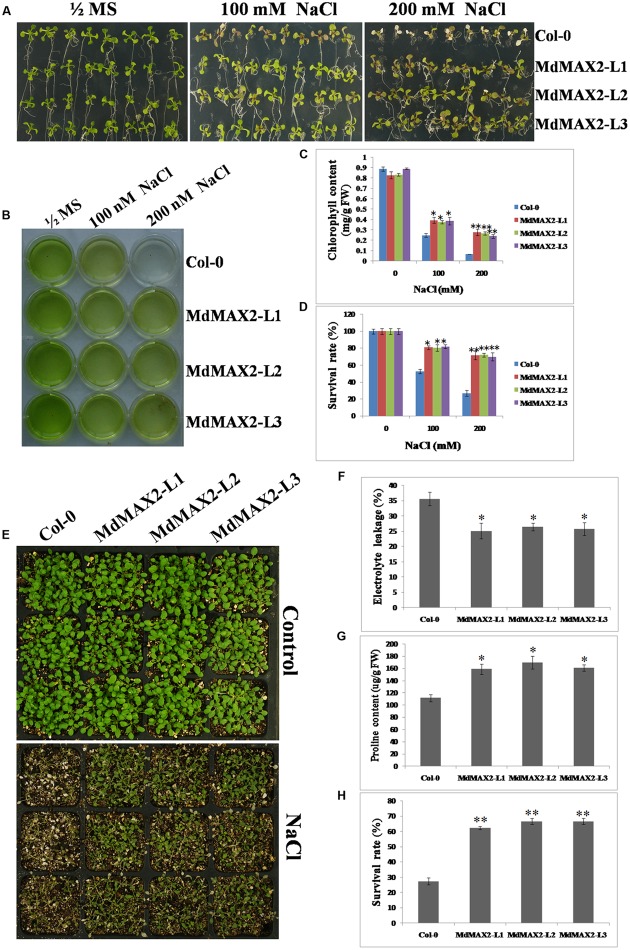
**Salt tolerance of *MdMAX2* transgenic *Arabidopsis*.** **(A)** The seedlings of wild-type (Col-0) and transgenic lines (L1, L2, and L3) germinated and grown on 1/2 MS medium supplemented with 0, 100, and 200 mM NaCl. Chlorophyll extraction solutions **(B)** and chlorophyll contents **(C)** in wild-type and transgenic plants. **(D)** Survival rates of wild-type and transgenic lines after NaCl treatment. **(E)** Phenotypes of 10-day-old transgenic *Arabidopsis* (MdMAX2-L1, MdMAX2-L2, and MdMAX2-L3) and the wild-type (Col-0) before and after 150 mM NaCl treatment for 7 days. **(F)** Electrolyte leakage and **(G)** proline content in Col-0 and *35S::MdMAX2* transgenic *Arabidopsis*. **(H)** Survival rates of wild-type and transgenic lines after 150 mM NaCl treatment. Values are the mean ± SD of three replica experiments, and asterisks denote Student’s test significance compared with the wild-type plants: ^∗^*P* < 0.05; ^∗∗^*P* < 0.01.

Overexpression of *MdMAX2* improved salt resistance in seedling germination; to determine whether it was also effective at the vegetable growth stage, 10-day-old wild-type *Arabidopsis* and *35S::MdMAX2* transgenic lines were transferred into 1/2 MS medium supplemented with 100 and 200 mM NaCl for 7 days. As shown in **Figure 6A**, the Col-0 seedlings showed wilting, but the transgenic seedlings remained green. Chlorophyll extracts (**Figure 6B**) and spectrophotometry analysis (**Figure 6C**) suggested that there were higher chlorophyll contents in the transgenic lines than in the wild-type seedlings. Additionally, higher survival rates (**Figure 6D**) further supported the role of MdMAX2 as a positive regulator of the plant response to salt stress.

Finally, *in vitro* salt tolerance assays of the transgenic plants and wild-type plants were conducted at the adult stage. Two-week-old individual genotype seedlings were grown on soil and sprayed with a NaCl solution for another 7 days. There were significant differences in the survival rates between the transgenic lines and the wild-type (**Figure 6E**), and the statistical survival rate analysis clearly verified the results (**Figure 6H**). Furthermore, the *35S::MdMAX2* transgenic *Arabidopsis* had significantly lower electrolyte leakage compared to the wild-type (**Figure 6F**). As shown in **Figure 6G**, the proline contents were higher in the three transgenic lines than in the wild-type after salt treatment. Finally, we also detected the expression levels of SOS-related genes (SOS1, SOS2, and SOS3) in wild-type control and transgenic lines, which were known for their key roles in salt tolerance (Ji et al., 2013). As expected, MdMAX2-overexpressing lines exhibited higher expression levels of SOS-related genes compared to wild-type control. All of the results demonstrated that the *MdMAX2*-overexpressing *Arabidopsis* conferred the tolerance to salt stress.

### Overexpression of *MdMAX2* in Apple Calli Enhanced the Tolerance to Salt Stress

**FIGURE 7:**
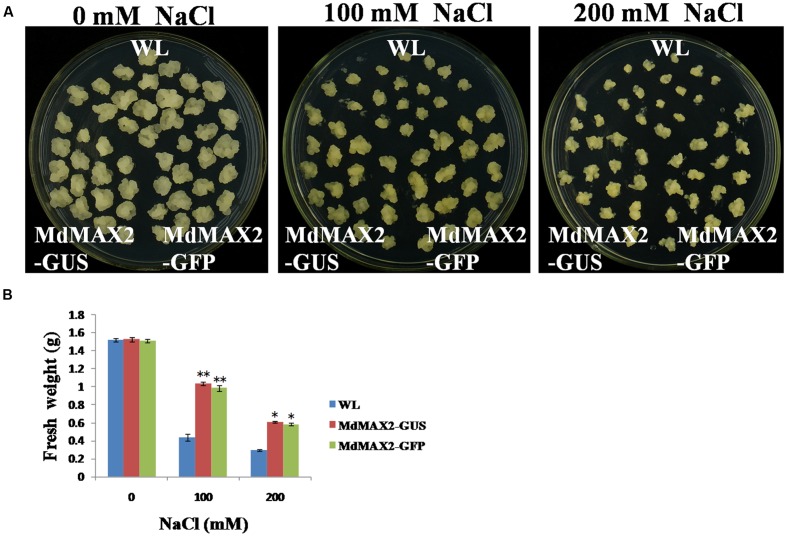
**Salt tolerance of *MdMAX2* transgenic apple calli.** **(A)** Apple calli of the indicated genotypes were grown in a medium with different concentrations of NaCl (0, 100, and 200 mM) for 10 days. **(B)** Fresh weight of wild-type (WL) and transgenic apple calli (MdMAX2-GUS and MdMAX2-GFP) after NaCl treatment. Values are the mean ± SD of three replica experiments, and asterisks denote Student’s test significance compared with the wild-type plants: ^∗^*P* < 0.05; ^∗∗^*P* < 0.01.

In addition, *35S::MdMAX2-GUS* and *35S::MdMAX2-GFP* transgenic apple calli were used for the salt stress test. The 10-day-old individual genotype calli were cultured on different concentrations of NaCl (0, 100, and 200 mM). Subsequently, these calli were grown for another 10 days under continuous dark conditions. As shown in **Figures 7A,B**, the transgenic apple calli exhibited obvious differences in growth, and overexpression of *MdMAX2* also resulted in a salt tolerance phenotype in apple calli.

### *MdMAX2*-Overexpressing *Arabidopsis* Enhanced the ABA-Mediated Stomata Aperture

**FIGURE 8:**
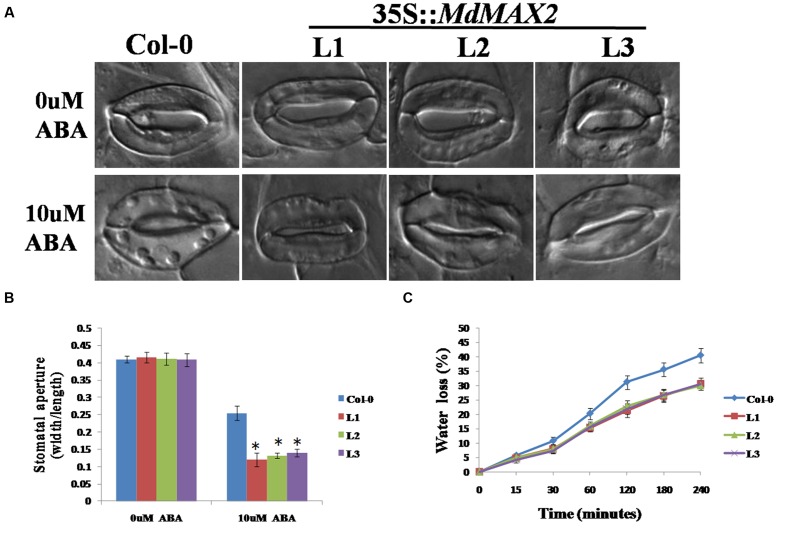
**MdMAX2 regulates abscisic acid (ABA)-mediated stomatal closure.** **(A)** Stomatal closure of the Col-0 and transgenic *Arabidopsis* (MdMAX2-L1, MdMAX2-L2, and MdMAX2-L3) in response to ABA. Rosette leaves of 2-week-old Arabidopsis of different genotypes were treated with stomatal opening solution for 2 h, and then incubated with 0 and 10 μM ABA for 2 h. **(B)** Stomatal aperture was calculated by ratios of width to length. **(C)** Water loss rates of 3-week-old *Arabidopsis* of different genotypes were measured at the indicated times. The water loss rate was expressed as the ratio between water loss and plant initial fresh weight. Values are the mean ± SD of three replica experiments, and asterisks denote Student’s test significance compared with the wild-type plants: ^∗^*P* < 0.05; ^∗∗^*P* < 0.01.

The results described above showed that MdMAX2 was responsive to ABA treatment (**Supplementary Figure S3B**). ABA is known to be an important phytohormone that is involved in multiple aspects of plant growth and development, and stomatal closure is an important ABA-medicated process that contributes to the maintenance of moisture under drought stress. To investigate the effect of MdMAX2 in the ABA-mediated sensitivity of guard cells, the stomatal apertures were measured in the Col-0 and three transgenic lines under ABA treatment. The 3-week-old rosette leaves of individual genotype lines were first pretreated with a stomatal opening solution and subsequently treated with 10 μM ABA for 3 h. The *MdMAX2*-overexpressing *Arabidopsis* displayed a smaller stomata aperture than the Col-0 plants treated with 10 μM ABA (**Figures 8A,B**). The results demonstrated that overexpression of *MdMAX2* was more sensitive to ABA in stomata closure. Under drought stress, stomata often close to limit water loss. Therefore, water loss assays were performed using detached leaves. The results revealed that lower water loss was detected in the *35S::MdMAX2* transgenic plants relative to Col-0 under drought stress treatment (**Figure 8C**). Taken together, these results indicated the possibility that MdMAX2 enhanced drought tolerance via decreased rates of water loss.

### *MdMAX2*-Overexpressing *Arabidopsis* Exhibits Increased Tolerance to Drought Stress

**FIGURE 9:**
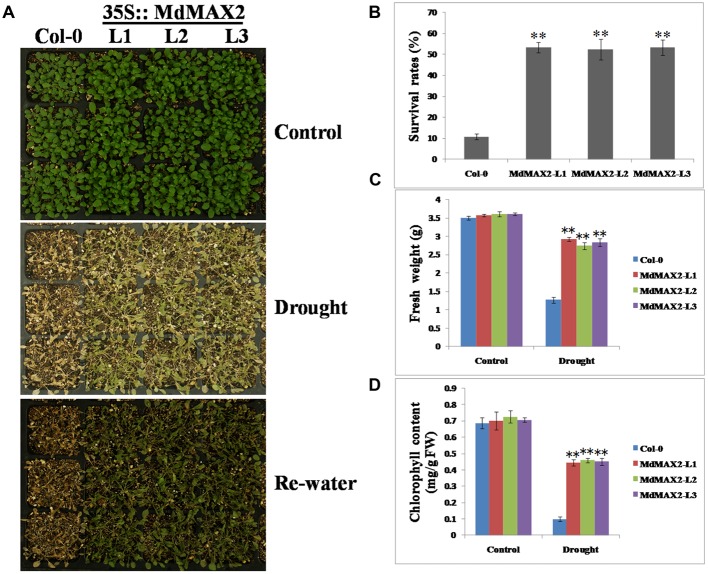
**Drought tolerance assays of *MdMAX2* transgenic *Arabidopsis*.** **(A)** Phenotypes of 10-day-old transgenic *Arabidopsis* (MdMAX2-L1, MdMAX2-L2, and MdMAX2-L3) and the wild-type (Col-0) after withholding water for 0 and 10 days, and then re-watering for 3 days. **(B)** Percent survival rates of Col-0 and transgenic plants. **(C)** Fresh weight, and **(D)** chlorophyll contents of the aboveground parts of transgenic or wild-type plants. Values are the mean ± SD of three replica experiments, and asterisks denote Student’s test significance compared with the wild-type plants: ^∗^*P* < 0.05; ^∗∗^*P* < 0.01.

To test the possible response of *MdMAX2*-overexpressing *Arabidopsis* to drought resistance, 2-week-old wild-type and *35S::MdMAX2* transgenic lines were subjected to water deficiency for 7 days. As shown in **Figures 9A,B**, wild-type plants wilted more rapidly than transgenic plants under continuous drought conditions. Additionally, wild-type plants exhibited more serious wilting symptoms and higher death rates compared with transgenic lines after re-watering for 2 days. Moreover, the fresh weight and chlorophyll contents were quantitatively investigated, and the results were consistent with our hypothesis (**Figures 9C,D**): overexpression of *MdMAX2* resulted in a phenotype of enhanced resistance to drought stress, including a heavier weight and higher chlorophyll content. These results suggested that MdMAX2 may play a key role in the drought tolerance response by mediating stomata aperture.

## Discussion

As a newly discovered plant hormone, SL plays an important role in perception and signal transduction to external environmental factors, thus making the plant adaptable to variable environments (Yoneyama et al., 2009). An F-box LRR protein, MAX2, functions as the receptor in the SL signaling pathway. Recently, many researches have investigated the functions of MAX2; an increasing number of studies have indicated that MAX2 regulates multiple developmental processes in plants, which are involved in leaf senescence, shoot branching (Wang et al., 2013), photomorphogenesis (Shen et al., 2012), and stress response (Van Ha et al., 2014). Among these responses, photomorphogenesis and stress response are economically important characteristics of plant configuration, stress-resistance cultivation, and increased yield in apple production. In this study, we isolated a gene with a highly conserved F-box motif in the N-terminus as well as two representative LRR motifs in the C-terminus that had a highly homologous relationship with other MAX2 proteins; this indicated that MdMAX2 might have similar functions to other MAX2 proteins (Waters and Smith, 2013; Wang et al., 2013). This study showed that MdMAX2 was involved in photomorphogenesis and stress response in apple.

### The Apple F-box Protein MdMAX2 Regulates Photomorphogenesis

Photomorphogenesis and skotomorphogenesis are important survival strategies in plant growth and development (Josse and Halliday, 2008; Sheerin et al., 2015). It is well documented that several phytohormones, such as Gibberellins, auxin, and Brassinosteroids (BRs), are involved in the photomorphogenesis and skotomorphogenesis processes (Tanaka et al., 2003; Alabadí et al., 2004). SLs are not involved in photomorphogenesis, whereas the SL signaling regulator MAX2 can promote photomorphogenesis by cooperating with multiple hormones (Shen et al., 2012). In this study, MdMAX2 was verified to be involved in photomorphogenesis in apple. Overexpression of *MdMAX2* promotes anthocyanin accumulation in apple calli and transgenic *Arabidopsis*. In addition, transgenic *Arabidopsis* exhibited a decreased hypocotyl length by shortening hypocotyl cells (**Figures 2** and **3**). These results suggest the MdMAX2 is a positive regulator of photomorphogenesis in apple.

### MdMAX2 Affects Photomorphogenesis Particularly through Regulating Auxin Homeostasis

Auxin was considered to be a vital player in anthocyanin accumulation and hypocotyl elongation (Gray et al., 1998; Jaakola, 2013). Additionally, increased auxin transport was responsible for the seedling de-etiolation phenotypes of a *max2* mutant (Shen et al., 2012). A hypothesis that auxin participated in *MdMAX2*-regulated photomorphogenesis was proposed. On the one hand, the transcripts of auxin transport-related genes and geotropism reaction assays revealed that auxin signaling may be altered by MdMAX2. On the other hand, DR5 activity was decreased in *35S::MdMAX2* transgenic plants, whereas there were no significant differences in the transcripts of auxin transport biosynthesis genes; these results indicated that auxin biosynthesis might also play a vital role in *MdMAX2*-regulated growth and physiological responses in accordance with other hormones.

### MdMAX2 Is Involved in Salt and Drought Stresses

Previous reports have demonstrated that SLs cooperatively regulated tolerance to multiple stresses with various phytohormones, such as ABA, brassinosteroid, and cytokinin (Van Ha et al., 2014). As an important regulator in the SL signaling network, MAX2 plays a vital role in the plant response to abiotic stress conditions. The *max2* mutants exhibited a phenotype of hypersensitivity to drought and salt stresses in *Arabidopsis* (Bu et al., 2014), indicating the positive regulatory role of MAX2 in the plant response to drought and salt stresses. In the present report, the functions of MdMAX2 in response to abiotic stress emerged to be probed into. The expression pattern of *MdMAX2* implied that it may be involved in the stress response (**Figures 5A–B**; **Supplementary Figure S3B**). Indeed, ectopic expression of *MdMAX2* in *Arabidopsis* improved the expression levels of SOS-related genes (**Supplementary Figure S3C**) and enhanced the tolerance to salt and drought stresses during both the seed germination stage and vegetable growth stage (**Figures 5**, **6**, and **9**). In addition, *MdMAX2* transgenic apple calli exhibited a similar salt-resistant phenotype. ABA is an essential mediator in both biotic and abiotic stress signaling pathways, and ABA-triggered-stomatal closure contributes to reducing water loss (Shinozaki and Yamaguchi-Shinozaki, 2007; Savchenko et al., 2014). This survival strategy is no exception in SL-mediated stress tolerance. The stomata of transgenic *MdMAX2 Arabidopsis* became sensitive to ABA treatment, and the water loss was significantly reduced (**Figure 8**); these data inferred that MdMAX2 played a conservative role with AtMAX2 in the salt and drought stress tolerance by regulating ABA-mediated stomata movements. Additionally, our data suggested that overexpression of *MdMAX2* had no impact on the development of root hair, despite some studies demonstrating that the *max2* mutant affected root hair elongation (**Supplementary Figure S4**) (Kapulnik et al., 2011). These results suggested that MdMAX2 might have distinct functions with *Arabidopsis* MAX2.

## Conclusion

MdMAX2 may act as a positive regulator in photomorphogenesis and the stress response and can potentially be used for plant configuration, stress improvement, and increased yield in apple as well as other species in the future.

## Author Contributions

Y-JH, X-FW, and J-PA designed the research. J-PA and X-FW performed the experiments. RL, F-JQ, and C-XY analyzed the data. Y-JH, J-PA, and X-FW wrote the manuscript text.

## Conflict of Interest Statement

The authors declare that the research was conducted in the absence of any commercial or financial relationships that could be construed as a potential conflict of interest.

## Footnotes

1 http://www.ncbi.nlm.nih.gov/BLAST/

2 http://smart.embl-heidelberg.de/

3 http://imagej.nih.gov/ij

4 http://rsbweb.nih.gov/ij

## Supplementary Material

**FIGURE S1 | Protein alignment for MdMAX2 and its homologs.** Alignment was performed by DNAMAN. The F-box domain and the LRR domains are indicated with red and blue marks, respectively.

**FIGURE S2 | Identification of *MdMAX2* transgenic apple calli and *Arabidopsis*.** **(A)** qRT-PCR identification of *MdMAX2*-overexpressing transgenic apple calli (MdMAX2-GUS and MdMAX2-GFP) and wild-type calli (WL). The value for WL was set at 1. **(B)** qRT-PCR identification of *MdMAX2*-overexpressing transgenic *Arabidopsis* lines (L1, L2, and L3) and wild-type *Arabidopsis* (Col-0). The value for Col-0 was set at 1. Values are the mean ± SD of three replica experiments, and asterisks denote Student’s test significance compared with the wild-type plants: ^∗^*P* < 0.05; ^∗∗^*P* < 0.01.

**FIGURE S3 | Expression patterns of *MdMAX2*.** **(A)** Expression of the *MdMAX2* gene in different organs of apple plants. Total RNA was isolated from various organs (roots, stems, leaves, flowers, and fruits) of adult plants. **(B)** Real-time PCR analysis of the expression of *MdMAX2* in response to abiotic stresses and plant hormones, including 150 mM NaCl, 300 mM Mannitol, 150 μM ABA, 150 μM GA, and 6% Glucose treatments. ‘Gala’ tissue cultures were treated in different conditions for 6 h and were collected for gene expression analysis. **(C)** Expression levels of SOS-related genes (SOS1, SOS2, and SOS3) in wild-type plants (Col-0) and transgenic lines. Values are the mean ± SD of three replica experiments, and asterisks denote Student’s test significance compared with the wild-type plants: ^∗^*P* < 0.05; ^∗∗^*P* < 0.01. **(D)** Expression levels of ARF-related genes (ARF1, ARF2, and ARF5) and other auxin biosynthesis genes (CYP798B2 and CYP798B3) in wild-type plants (Col-0) and transgenic lines. Values are the mean ± SD of three replica experiments, and asterisks denote Student’s test significance compared with the wild-type plants: ^∗^*P* < 0.05; ^∗∗^*P* < 0.01.

**FIGURE S4 | Effect of MdMAX2 on length and density of root hairs.** **(A)** Representative primary root from wild-type (Col-0) and transgenic *Arabidopsis* (MdMAX2-L1, MdMAX2-L2, and MdMAX2-L3) at 4 days after germination (DAG). Root hair length **(B)** and density **(C)** are shown. Three independent experiments were performed, involving 150–200 root hairs for length measurement and 30–40 roots for hair density.
