# Supplementary figures and images for: Apple F-Box Protein MdMAX2 Regulates Plant Photomorphogenesis and Stress Response
Source: Front Plant Sci. 2016 Nov 17;7:1685. doi: 10.3389/fpls.2016.01685 (PMC5112277; doi:10.3389/fpls.2016.01685)

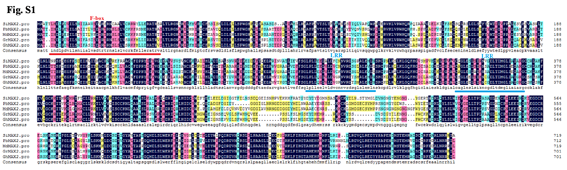

Supplement: Supplementary file 1 [file Image_1.TIF]

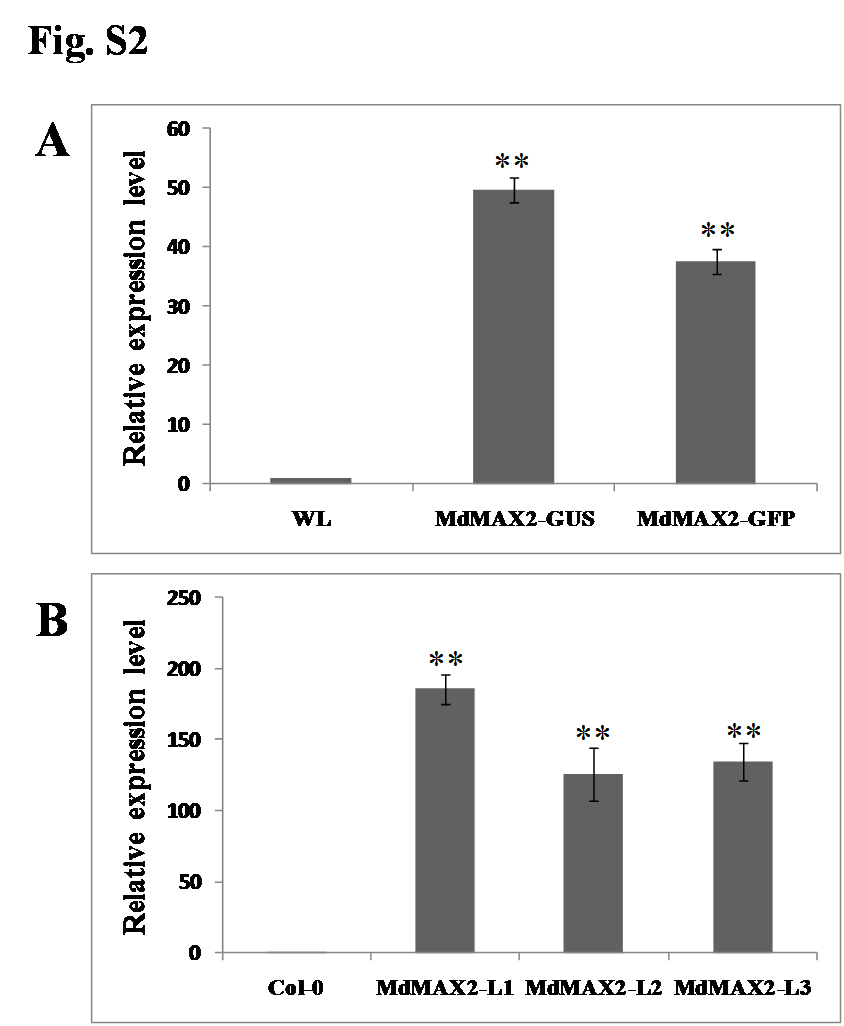

Supplement: Supplementary file 2 [file Image_2.TIF]

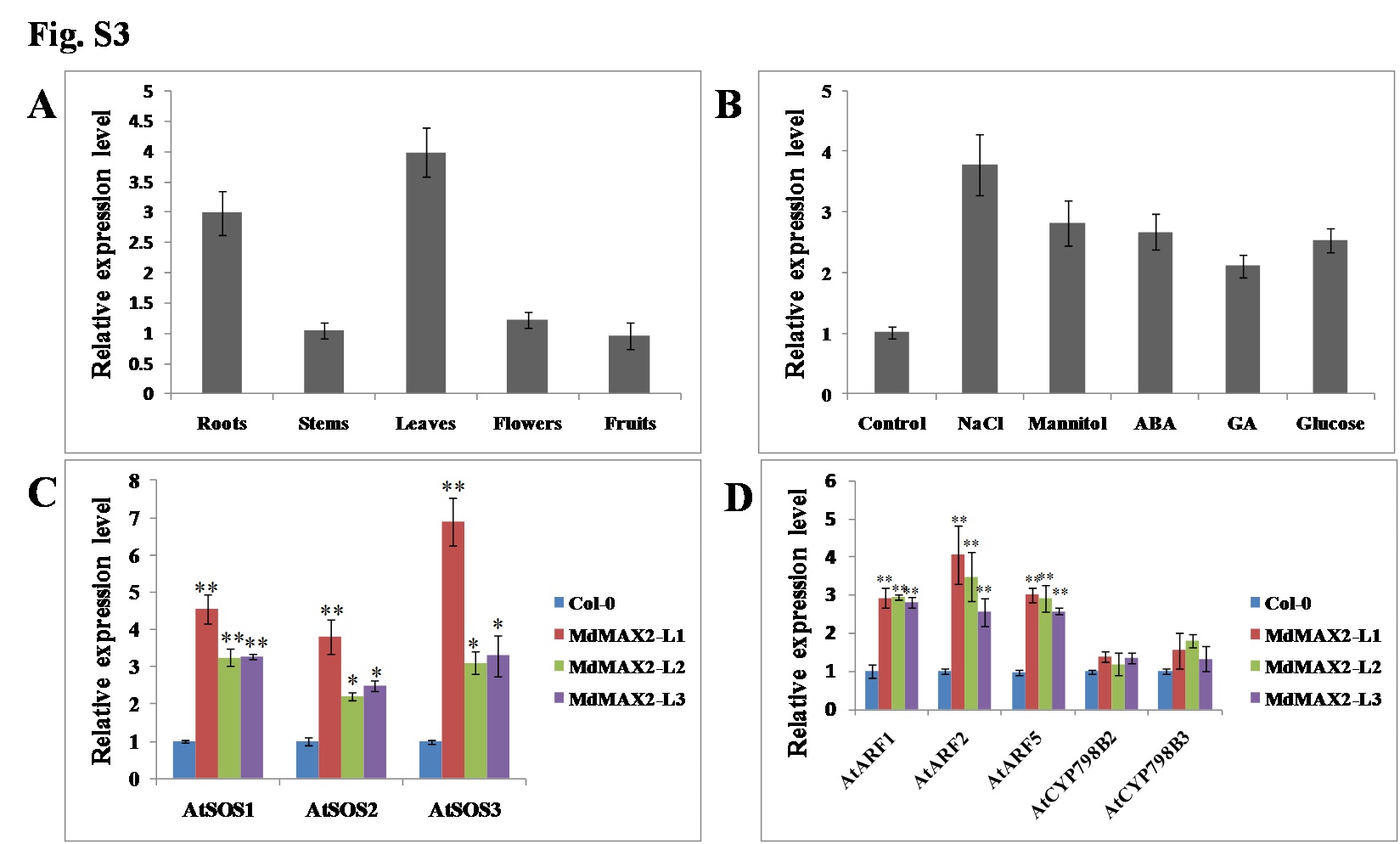

Supplement: Supplementary file 3 [file Image_3.JPEG]

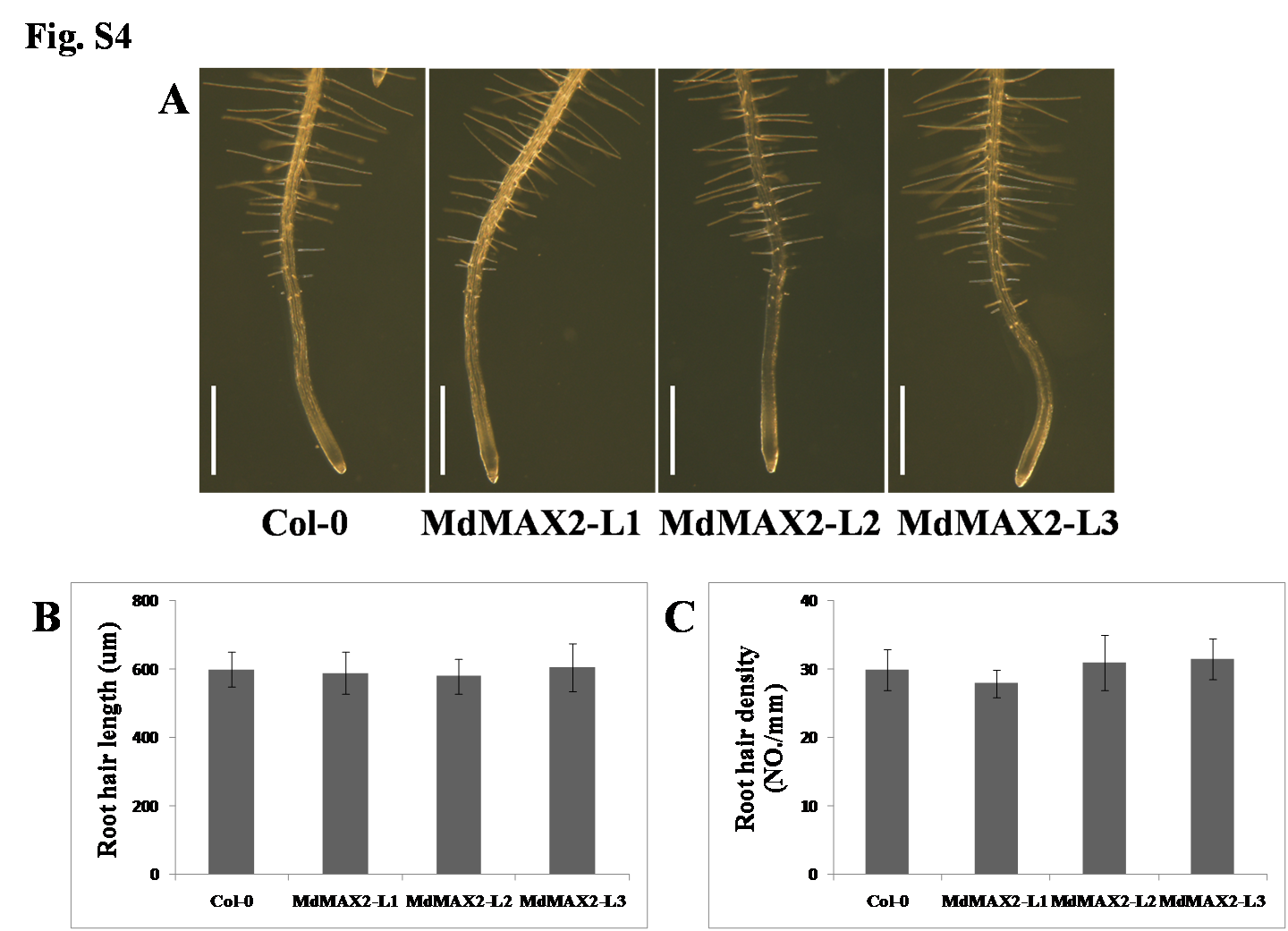

Supplement: Supplementary file 4 [file Image_4.TIF]
