# Supplementary material for: Apple F-Box Protein MdMAX2 Regulates Plant Photomorphogenesis and Stress Response
Source: Front Plant Sci. 2016 Nov 17;7:1685. doi: 10.3389/fpls.2016.01685 (PMC5112277; doi:10.3389/fpls.2016.01685)
Supplement: Supplementary file 5 [file Table_1.DOCX]

**Supplementary Table 1**

Primers used for gene expression analysis and vector construction in this study.

Primer name sequence (5’ to 3’)

MdMAX2-F: CTCACTCTCACTCATTCCCTC

MdMAX2-R: CTCGTACATTGTTCCTCTCCG

MdMAX2(qRT)-F: AGCTACCATAACTCCTCCCT

MdMAX2(qRT)-R: TCTCCATGCTCCGGAACTT

MdDFR(qRT)-F: GTTGAGGGAGATAGGGTTTGAG

MdDFR(qRT)-R: GGTAAATGTAAAACAATAGAGAGG

MdUFGT(qRT)-F: GGAAGTGGTTTTGTCGCCTG

MdUFGT(qRT)-R: CATTATTATTGAGCAACGAACAGC

MdF3H(qRT)-F: GCCGATCACCTACACCGAG

MdF3H(qRT)-R: GTACAAGAAGTGGGAAGGC

MdCHI(qRT)-F: GCTACAAATGCGGTGATAG

MdCHI(qRT)-R: CGCCTCCACTACAACCTCC

MdCHS(qRT)-F: GGCAAGTGCTGTCGGATT

MdCHS(qRT)-R: CCCAAAGAAATAACCACAAG

MdANR(qRT)-F: TCAACAAAAGATACCCCCAG

MdANR(qRT)-R: GATAGCTAGCTCGATACATGC

AtCHI(qRT)-F: CCGGTTCATCGATCCTCTTC

AtCHI(qRT)-R: ATCCCGGTTTCAGGGATACTATC

AtCHS(qRT)-F: CGCATCACCAACAGTGAACAC

AtCHS(qRT)-R: TCCTCCGTCAGATGCATGTG

AtDFR(qRT)-F: CCTTATCACCGCGCTCTCT

AtDFR(qRT)-R: TGTCCTTGTCTTATGATCGAGTAATGC

AtUF3GT(qRT)-F: CAACTGGTTTTCCGTTTCTGGTT

AtUF3GT(qRT)-R: GCTTCCTCGACGGTTGATACAC

AtPAL(qRT)-F: GCTTCCGAATATTCCGGCGTTAA

AtPAL(qRT)-R: CCAAAAACGGTGTCGCACT

AtAUX1(qRT)-F: TTGGTTCAGCTGCGCATCTA

AtAUX1(qRT)-R: GCAGTCCAGCTTCCTAGTAA

AtPIN1(qRT)-F: CGGTGGGAACAACATAAGCA

AtPIN1(qRT)-R: CACACTTGTTGGTGGCATCAC

AtPIN2(qRT)-F: CCGTGGGGCTAAGCTTCTCATCT

AtPIN2(qRT)-R: AGCTTTCCGTCGTCTCCTATCTCC

AtPIN3(qRT)-F: CGGAGCACCTGACAACGAT

AtPIN3(qRT)-R: CGGATCTCTTTAGCACCTTGGT

AtPIN4(qRT)-F: GTTGCTGGGATTGCCATTG

AtPIN4(qRT)-R: GCAGCCTGAACGATGGCTAT

AtPIN7(qRT)-F: CATAAACCGCTTCGTCGCTAT

AtPIN7(qRT)-R: TTGAGGAGATGAAGTGGAAAGAGA

AtYUC1(qRT)-F: GCATCGCTCCAAGGTTCAAC

AtYUC1(qRT)-R: ACTTCTTTCTCCCTGGAATCTCG

AtYUC2(qRT)-F: CAAGGTGTATCCGGAGTTGA

AtYUC2(qRT)-R: AATGGCTGCACCAAGCAATC

AtYUC4(qRT)-F: ACTCCATCTCCCTAAACAC

AtYUC4(qRT)-R: CTTGCGTCTTCACATTCC

AtYUC6(qRT)-F: TATACGCGGTCGGATTCACA

AtYUC6(qRT)-R: CCACCACAATCACTCTCAT

AtTAA1(qRT)-F: GATGAAGAATCGGTGGGAGAAGC

AtTAA1(qRT)-R: CGTCCCTAGCCACGCAAACGCAGG

AtSOS1(qRT)-F: AGTGTAAGTTTCGGTGGGATC

AtSOS1(qRT)-R: CACGCATGTTTACGGGTTTC

AtSOS2(qRT)-F: CGATCAAGGCCGGACAGTTA

AtSOS2(qRT)-R: TCCTTCTGTTGCCCTCCATA

AtSOS3(qRT)-F: ATGATTGAAGTAATGGTGGATAAGG

AtSOS3(qRT)-R: ATGAAACGAAACTTGGAAACG

AtARF1(qRT)-F: CGGATAAGCTTGGTCTCC

AtARF1(qRT)-R: TTGGTCACCTTCAGCTTG

AtARF2(qRT)-F: CCTCATCCGAAGGATGCTCAAACG

AtARF2(qRT)-R: GGAGCCATCAACTCTCCATTGAACTC

AtARF5(qRT)-F: CGGGAAACGAAGGTACATGGGAAC

AtARF5(qRT)-R: TACGCCACTTAGAACCAGGCCATC

AtCYP798B2(qRT)-F: CAGAGACAACAGAAACCACAA

AtCYP798B2(qRT)-R: TTGAGAGGAGATACAAGGTGCT

AtCYP798B3(qRT)-F: CTTTGCGTCAAGACCACTC

AtCYP798B3(qRT)-F: CTATTGTCGTGTAGCCAACG
